# Retrospective analysis of risk factors for postoperative perineal hernia after endoscopic abdominoperineal excision for rectal cancer

**DOI:** 10.1186/s12893-022-01538-7

**Published:** 2022-03-08

**Authors:** Tatsuya Manabe, Yusuke Mizuuchi, Yasuhiro Tsuru, Hiroshi Kitagawa, Takaaki Fujimoto, Yasuo Koga, Masafumi Nakamura, Hirokazu Noshiro

**Affiliations:** 1 Department of Surgery, Faculty of Medicine, Saga University, 5-1-1 Nabeshima, Saga, 849-8501 Japan; 2 Department of Surgery and Oncology, Graduate School of Medical Sciences, Kyushu University, 3-1-1 Maidashi, Higashi-ku, Fukuoka, 812-8582 Japan

**Keywords:** Postoperative perineal hernia, Endoscopic abdominoperineal excision, Rectal cancer

## Abstract

**Background:**

In contrast to open-surgery abdominoperineal excision (APE) for rectal cancer, postoperative perineal hernia (PPH) is reported to increase after extralevator APE and endoscopic surgery. In this study, therefore, we aimed to determine the risk factors for PPH after endoscopic APE.

**Methods:**

A total 73 patients who underwent endoscopic APE for rectal cancer were collected from January 2009 to March 2020, and the risk factors for PPH were analyzed retrospectively.

**Results:**

Nineteen patients (26%) developed PPH after endoscopic APE, and the diagnosis of PPH was made at 9–393 days (median: 183 days) after initial surgery. Logistic regression analysis showed that absence of pelvic peritoneal closure alone increased the incidence of PPH significantly (odds ratio; 13.76, 95% confidence interval; 1.48–1884.84, *p* = 0.004).

**Conclusions:**

This preliminary study showed that pelvic peritoneal closure could prevent PPH after endoscopic APE.

## Background

Postoperative perineal hernia (PPH) after abdominoperineal excision (APE) of the rectum is a complication caused by herniation of the intra-abdominal organs through the pelvic floor after complete removal of the anorectal sequence. Although most PPHs after APE are asymptomatic or ignorable, some patients have serious symptoms such as discomfort, perineal pain, impaired sensation, urinary dysfunction or intestinal obstruction when perineal bulging is gradually enlarged [1, 2]. Therefore, some patients have disturbed quality of life and others require surgical treatment. In patients with conventional open APE, the incidence of clinically manifest PPH was reported as < 1% [3, 4] and PPH based on barium X-rays was 7% [5]. However, recent technical modifications in APE for rectal cancer are associated with increased incidence of PPH. One such modification is extralevator APE (ELAPE) for rectal cancer, which involves wide resection of the levator ani muscles surrounding the rectum through two-phase abdominal and perineal resection to obtain sufficient circumferential resection margins and prevent inadvertent rectal rupture [6]. Despite the improved oncological outcomes, increased perineal complications have been reported after removal of excessive pelvic tissue in ELAPE, compared with conventional APE [7–10]. To prevent PPH, therefore, exact pelvic reconstruction, such as the myocutaneous flap method or use of a biological mesh, has been performed after ELAPE [6, 11, 12]. In contrast, endoscopic surgery is associated with reduced incidence of ventral hernia after colorectal surgery [13], but an increased incidence of PPH after endoscopic APE has been reported [9]; thus, some preventive procedure against PPH is advocated.

In previous studies, risk factors for PPH after conventional open APE included previous hysterectomy, perineal wound infection, perioperative radiotherapy, coccygectomy, excessive length of small bowel mesentery, and larger size of the female pelvis [3, 14–17]. However, most of these reports were from small studies or case reports, and the risk factors for PPH after endoscopic APE for rectal cancer are not well documented until now. In this study, we conducted retrospective analysis to clarify the incidence and risk factors for PPH after endoscopic APE for rectal cancer.

## Methods

**Fig. 1:**
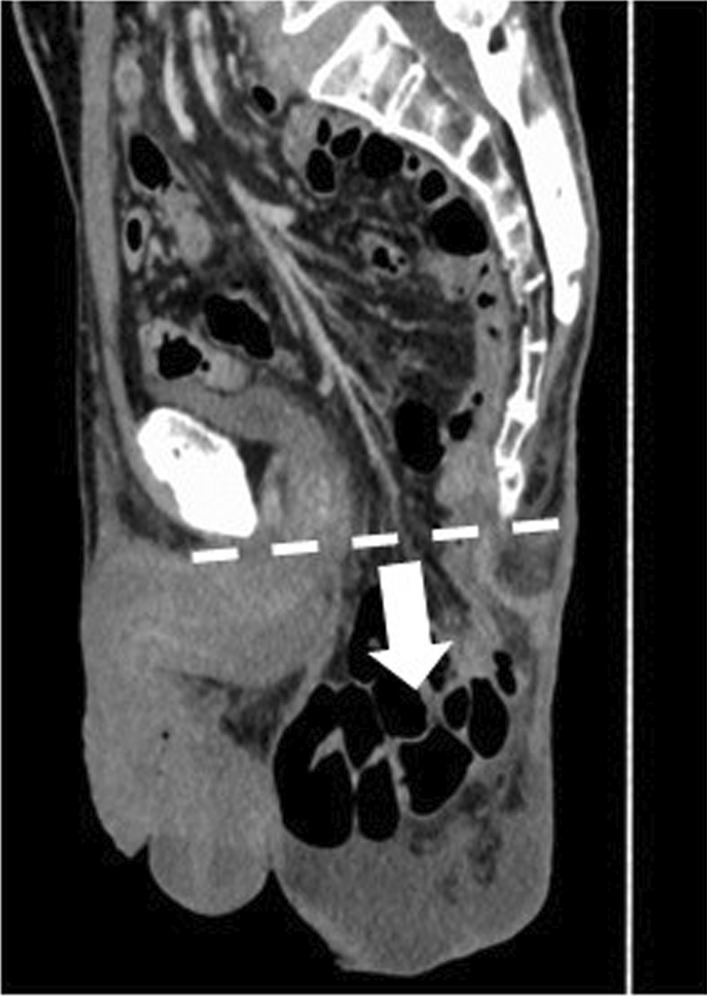
Diagnosis of postoperative perineal hernia by computed tomography is defined as the downward displacement of the intestine beyond the line described by computed tomography from the inferior margin of the pubis to the end of the coccyx

A total of 75 patients with rectal cancer underwent endoscopic APE with simple closure of the perineum at Saga University Hospital or the Department of Surgery and Oncology in Kyushu University Hospital between January 2009 and March 2020. Patients who underwent total pelvic exenteration were excluded. PPH was defined as an obvious bulge in the perineum and/or downward displacement of the intestine beyond the line described by computed tomography from the inferior margin of the pubis to the end of the coccyx (Fig. 1). Standard surveillance using computed tomography was routinely carried out every 6 months for at least 5 years after surgery, and irregularly performed to investigate other disease, based on the physician’s decision.

The demographics of the patients were obtained from the prospectively maintained comprehensive database and medical records. The tumor stage was classified according to the eighth TNM classification system. Patients with clinical T4, pelvic nodal involvement and/or circumferential resection margin < 1 mm by magnetic resonance imaging received preoperative chemoradiotherapy and/or systemic chemotherapy. Postoperative systemic chemotherapy was administered to patients with pathologically positive lymph nodes and/or distant metastases. Patient-related variables, tumor-related variables, therapeutic variables and postoperative variables were investigated to clarify the risk factors for PPH.

### Ethics

All procedures in this study were conducted in accordance with the ethical standards of the responsible committee on human study and with the Helsinki Declaration and later revision. The demographics of the patients were obtained from the prospectively maintained comprehensive database and the medical records. Informed consent for the use of medical information was obtained from all patients. The study protocol was approved by the Ethics Committee of the Faculty of Medicine at Saga University (2019-09-Jinsoku-03) and Kyushu University (29-292).

### Endoscopic APE

**Fig. 2:**
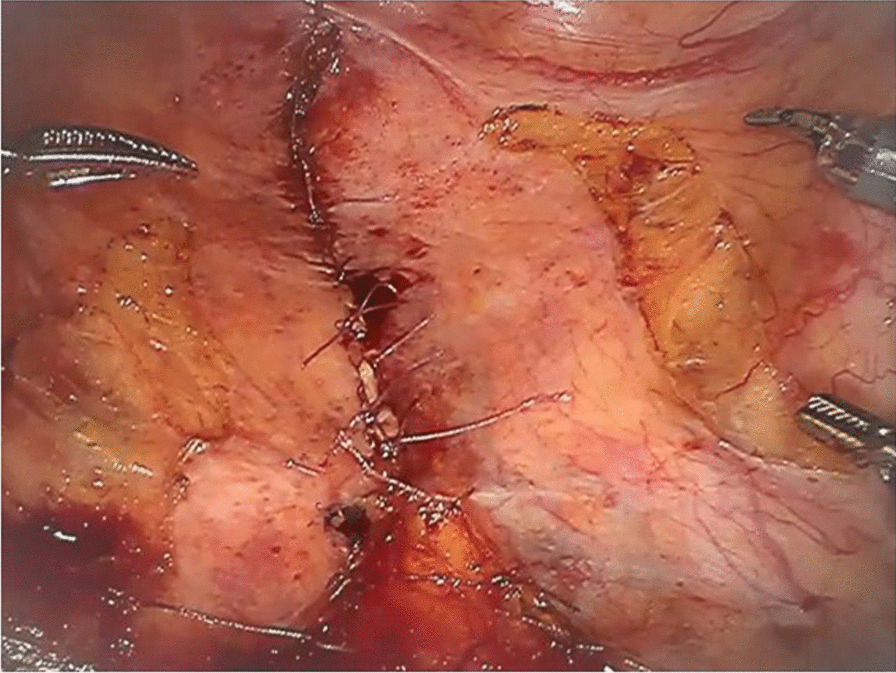
Endoscopic view of pelvic peritoneal closure with shallow incision

All patients were placed in the supine modified Lloyd–Davies position. Laparoscopic surgery was performed using a five-port technique: a supra-umbilical port for the laparoscope, two ports at the right lower quadrant, and two ports placed symmetrically at the left lower quadrant. For robot-assisted APE using the da Vinci Si Surgical System (Intuitive Surgical Inc., Sunnyvale, CA, USA), six ports were placed as described previously [18]. Typically, after ligation of the inferior mesenteric artery, mobilization of the rectum with total mesorectal excision (TME) preserving the autonomic nerves was performed in the pelvis along the presacral space. Posterior dissection in the TME plane stopped at the apex of the coccyx. Next, the lateral ligaments were divided bilaterally and the peritoneal reflection was opened, and the anterior side of the rectum was dissociated to the lower edge of the prostate for men, or along the rectovaginal septum for women. The levator ani muscle was divided transabdominally from the posterior to lateral side to the ischiorectal fossa. When endoscopic transperineal TME was performed, vascular ligation and dissection of the upper rectum were laparoscopically performed and the levator ani muscle was divided via the perineal approach. Finally, the specimen was extracted through the perineal wound. Closure of the perineum was performed by primary approximation of the skin and subcutaneous tissue. PPC was added for some patients. After specimen removal, the pelvic peritoneum was closed neatly with interrupted 3–0 Vicryl sutures from the anterior to posterior under laparoscopic vision and/or using robotic arms (Fig. 2). The choices of surgical approach, route of the stoma, and PPC depended on the discretion of the treating surgeon.

### Statistical analysis

All statistical analyses were performed using JMP version 14 (SAS Institute, Cary, NC, USA). For descriptive analysis, continuous variables were compared between the groups by the Mann–Whitney U test, while the chi-squared test and analysis of variance were used for comparison of categorical variables. For univariate analysis, simple logistic regression analysis was used. Multiple logistic regression analysis with Firth correction was performed to identify factors that were independently associated with PPH. *p* < 0.05 was considered to be statistically significant.

## Results

**Fig. 3:**
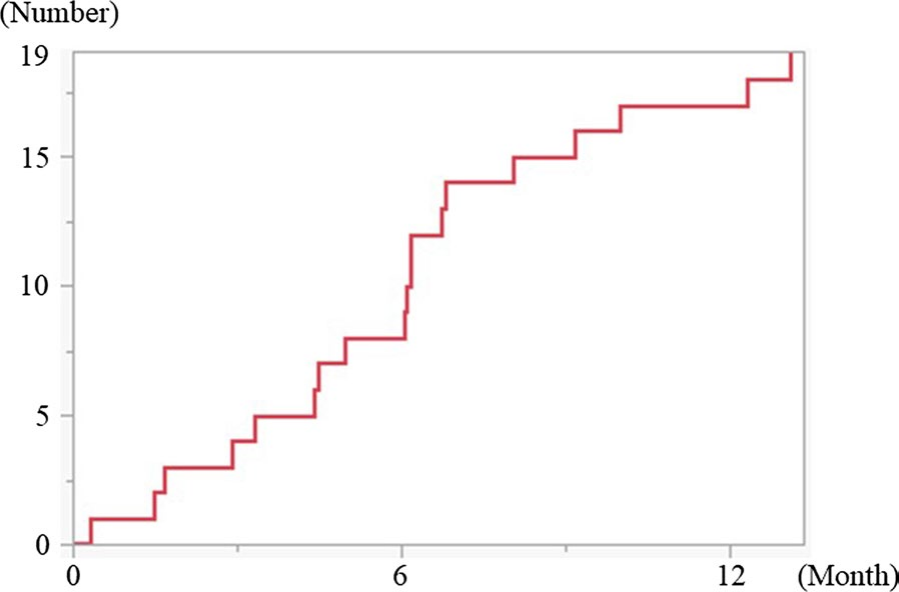
Cumulative number of patients with postoperative perineal hernia after endoscopic abdominoperineal excision

Patient, tumor, therapeutic and postoperative characteristics are summarized in Table 1. Median observation period was 963 days (range 9–2190 days). Of all 73 patients who underwent endoscopic APE for rectal cancer, 19 (26%) developed PPH. Three of these 19 patients received hernia repair for the severe symptoms. The cumulative incidence of PPH is shown in Fig. 3. The median period of detection of PPH was 183 days (range 9–393 days) after surgery. PPH did not occur in any patient > 2 years after surgery.

**Table 1:** Patient and Clinical Characteristics

Parameters	Total (n)	Perineal hernia		*p* value
		Presence	Absence	
		n = 19	n = 54	
Patient-related variables				
Age				
Median (range)		66 (42–89)	68 (31–86)	0.472
Sex				
Male	49	11	38	0.325
Female	24	8	16	
BMI* (kg/m^2^)				
Median (range)		20.3 (17.9–34.8)	21.5 (15.9–33.8)	0.991
ASA-PS**				
1	15	6	9	0.404
2	54	12	42	
3	4	1	3	
Tumor-related variables				
Depth of the tumor				
pT1	5	0	5	0.344
pT2	19	5	14	
pT3	40	10	30	
pT4b	7	3	4	
CR	2	1	1	
Maximum diameter of tumor (mm)				
Median (range)		47 (0–116)	44 (0–280)	0.173
Site of inferior margin of the tumor				
Upper rectum		0	2	0.387
Lower rectum		15	36	
Anal canal		4	16	
Simultaneous distant metastasis				
Yes	7	1	6	0.431
No	66	18	48	
Therapeutic variables				
Preoperative therapy				
Total				
Yes	21	7	14	0.373
No	62	12	40	
NCRT***				
Yes	9	4	5	0.200
No	64	15	49	
Systemic chemotherapy				
Yes	15	4	11	0.950
No	58	15	43	
Surgical approach				
Endoscopic surgery	56	14	42	0.738
Robot-assisted surgery	8	3	5	
Trans-perineal approach	9	2	7	
Multivisceral resection				
Yes	7	3	4	0.308
No	66	16	50	
Lateral pelvic lymphnode dissection				
Yes	37	9	28	0.737
No	36	10	26	
Route of stoma				
Transperitoneal route	51	14	37	0.670
Retroperitoneal route	22	5	17	
Pelvic peritoneal closure				
Yes	11	0	11	0.007
No	62	19	43	
Operating time (min)				
Median (range)		553 (276–850)	671 (281–1089)	0.256
Bleeding (g)				
Median (range)		210 (0–940)	232 (0–1267)	0.799
Transfusion				
Yes	10	2	8	0.632
No	63	17	46	
Residual tumor				
R0^#^	66	17	49	0.732
R1^##^	2	1	1	
R2^###^	5	1	4	
Postoperative systemic chemotherapy				
Yes	33	7	26	0.392
No	40	12	28	
Postoperative variables				
Postoperative complication				
Perineal wound dehiscence				
Yes	8	4	4	0.120
No	65	15	50	
Pelvic abscess				
Yes	9	1	8	0.240
No	64	18	46	
Urinary disorder				
Yes	12	3	9	0.929
No	61	16	45	
Ileus				
Yes	9	2	7	0.778
No	64	17	47	
Length of postoperative hospital stay (day)				
Median (range)		17 (8–66)	18 (5–75)	0.898

*BMI: body mass index, **ASA-PS: American Society of Anesthesiologists physical status, ***NCRT: neoadjuvant chemoradiotherapy, ^#^R0: resection for cure or complete remission, ^##^R1: microscopic residual tumor, ^###^R2: macroscopic residual tumor

Table 2 shows the results of univariate analysis. No patient-related, tumor-related or postoperative variables were associated with PPH. Among therapeutic variables, preoperative therapy, surgical approach, performance of transperineal endoscopic approach, addition of pelvic lymph node dissection, route of the stoma, operating time, blood loss volume, transfusion residual tumor, and postoperative systemic chemotherapy were not associated with PPH. PPC alone was significantly associated with the incidence of PPH (*p* = 0.012). Multivariate logistic regression analysis with Firth correction that included PPC and postoperative perineal wound dehiscence was performed to identify independent factors associated with occurrence of PPH. PPC was independently associated with the occurrence of PPH (odds ratio = 13.757, 95% confidence interval = 1.484–1884.838; *p* = 0.004) (Table 3).

**Table 2:** Univariate analysis to evaluate the risk factors for postoperative perineal hernia

Parameters	OR (95% CI)	*p* value
Patient-related variables		
Age	1.016 (0.974–1.059)	0.467
Sex		
Male/Female	0.5789 (0.1962–1.7081)	0.325
BMI*	1.0009 (0.8558–1.1706)	0.991
ASA-PS**		
1/2	2.3333 (0.6916–7.8719)	0.172
1/3	2.0000 (0.1662–24.0689)	0.585
2/3	0.8571 (0.0816–9.0087)	0.898
Tumor-related variables		
Depth of the tumor		
< pT2/> pT3	0.7846 (0.2575–2.3901)	0.67
Maximum diameter of tumor (mm)	0.9975 (0.9821–1.0132)	0.746
Site of inferior margin of the tumor		
Rectum/Anal canal	1.5479 (0.4533–5.5003)	0.473
Simultaneous distant metastasis	0.4444 (0.0500–3.9522)	0.467
Therapeutic variables		
Preoperative therapy		
Total	1.6667 (0.5475–5.074)	0.369
NCRT***	2.6133 (0.6214–10.9901)	0.19
Systemic chemotherapy	1.0424 (0.2880–3.774)	0.95
Surgical approach		
Endoscopic/Robot	0.5556 (0.1175–2.6277)	0.459
Endoscopic/Trans-perineal	1.1667 (0.2166–6.2840)	0.858
Robot/Trans-perineal	2.100 (0.2507–17.5941)	0.494
Multivisceral resection	2.344 (0.4735–11.6006)	0.297
Lateral pelvic lymphnode dissection	0.8357 (0.2934–2.3807)	0.737
Route of stoma		
Transperitoneal/Retroperitoneal	1.286 (0.3987–4.1514)	0.673
Pelvic peritoneal closure	3.2110 (0.1029–3.6031)	0.012
Operating time	0.2071 (0.0139–4.8280)	0.245
Bleeding	1.0002 (0.9983–1.0023)	0.794
Transfusion	0.6765 (0.1304–3.5095)	0.642
Residual tumor		
R0^#^/R1^##^	0.3469 (0.0206–5.857)	0.463
R0^#^/R2^###^	1.3878 (0.1449–13.2948)	0.776
R1^##^/R2^###^	4.0000 (0.1168–136.9573)	0.442
Postoperative systemic chemotherapy	0.6282 (0.2146–1.8391)	0.396
Postoperative variables		
Postoperative complication		
Perineal wound dehiscence	3.3333 (0.7429–149571)	0.122
Pelvic abscess	0.3194 (0.0372–2.7399)	0.298
Urinary disorder	0.9375 (0.2253–3.9009)	0.929
Ileus	0.7899 (0.1492–4.1814)	0.782
Length of postoperative hospital stay	1.0025 (0.9650–10415)	0.897

*BMI: body mass index, **ASA-PS: American Society of Anesthesiologists physical status, ***NCRT: neoadjuvant chemoradiotherapy, ^#^R0: resection for cure or complete remission, ^##^R1: microscopic residual tumor, ^###^R2: macroscopic residual tumor

**Table 3:** Multiple logistic regression analysis with Firth correction to determine independent risk factors for Postoperative perineal hernia

Parameters	OR (95% CI)	p value
Peritoneal closure of the pelvis	13.757 (1.484–1884.838)	0.004
Perineal wound dehiscence	0.211 (0.034–1.042)	0.057

## Discussion

This study showed that PPH occurred in 26% of patients with endoscopic APE for rectal cancer within 13 months after surgery and that PPC was available for prevention of PPH. The importance of PPC for preventing perineal complications was advocated by McMullin [4] and Goliger [19] in 1985. In conventional open APE, PPC is a standard procedure when sufficient peritoneal tissue is preserved [20]. Similarly, Yan et al. [21] reported that no PPH was found in 86 cases that underwent endoscopic APE with additional PPC, and that the incidence of PPH was significantly lower in endoscopic APE with than without PPC (0% vs 5.21%, *p* = 0.032). Nevertheless, the pelvic peritoneum is often not closed during endoscopic APE because laparoscopy is necessary for proficient suturing [22, 23]. In contrast to the previous reports about the risk factors for PPH [3, 14–17], this study did not show that PPH had any correlation with female sex, preoperative radiotherapy, or multiple organ resection including coccygectomy. Measurement of the mesenteric length was not accessible under the laparoscopic approach.

Although PPC is a useful technique to prevent PPH, some discussion remains before performing PPC. First, the peritoneum must be removed widely to avoid division of the mesorectum during medial and lateral dissection of the upper rectum from the pelvis under laparoscopy. When it is hard to perform peritoneal closure because of severe tension, addition of a shallow incision on the tense portion of the peritoneum could be helpful to relax it [21]. During suturing of the peritoneum, the stitching intervals should be shortened, because herniation of the intestine through the unexpected defect of the closed peritoneum could occur. Indeed, we did not observe herniation because interrupted stitches were placed at short intervals during peritoneal closure. Next, high proficiency is mandatory in suturing procedures by conventional laparoscopic surgery. Robotic surgery might facilitate such procedures. Finally, PPC could not be performed in some patients with endoscopic APE because of tumor invasion to the pelvic peritoneum, bulky tumor, addition of lateral pelvic lymph-node dissection, and preoperative chemoradiotherapy [24].

Various pelvic reinforcements as alternatives to PPC have been performed after APE: suture of levator ani muscle, bladder peritoneal flaps, hysteropexy, omentoplasty and synthetic mesh. Levator ani muscle suturing [25] could not be applied to rectal cancer surgery because of wide excision of the muscle. A randomized trial revealed that omentoplasty did not reduce the incidence of PPH [26]. Several studies have revealed that Bio-mesh can be effective for reducing PPH [10, 12, 27]. Unfortunately, the use of Bio-mesh is limited to western countries. Immobilization of bladder peritoneal flaps in men and the uterus in women might be helpful for preventing PPH, when PPC is impossible [28, 29].

The present study had some limitations: the retrospective design, small study population, and application of the approach for lateral pelvic lymph-node dissection and PPC was decided by surgeons. Therefore, this study data is preliminary, and a large number study would be needed to confirm this data.

## Conclusions

This preliminary study suggested that the only risk factor for PPH was absence of PPC. Therefore, PPC could prevent PPH after endoscopic APE for rectal cancer. A further study is needed to confirm the risk factor for PPH.

## Data Availability

The datasets used and analyzed during the current study available from the corresponding author on reasonable request.

## Abbreviations

APE: Abdominoperineal excision
PPH: Postoperative perineal hernia
PPC: Pelvic peritoneal closure
ELAPE: Extralevator APE
TME: Total mesorectal excision
